# Phred-Phrap package to analyses tools: a pipeline to facilitate population genetics re-sequencing studies

**DOI:** 10.1186/2041-2223-2-3

**Published:** 2011-02-01

**Authors:** Moara Machado, Wagner CS Magalhães, Allan Sene, Bruno Araújo, Alessandra C Faria-Campos, Stephen J Chanock, Leandro Scott, Guilherme Oliveira, Eduardo Tarazona-Santos, Maira R Rodrigues

**Affiliations:** 1 Departamento de Biologia Geral, Instituto de Ciências Biológicas, Universidade Federal de Minas Gerais, Av Antonio Carlos 6627, Pampulha, Caixa Postal 486, Belo Horizonte, MG, CEP 31270-910, Brazil; 2 Departamento de Ciência da Computação, Instituto de Ciências Exatas, Universidade Federal de Minas Gerais, Av Antonio Carlos 6627, Pampulha, Belo Horizonte, MG, CEP 31270-910, Brazil; 3 Laboratory of Translational Genomics of the Division of Cancer Epidemiology and Genetics, National Cancer Institute, National Institutes of Health, Gaithersburg, MD, USA; 4 8717 Grovemont Circle Advanced Technology Center, Room 127, Gaithersburg, MD, 20877, USA; 5 Genomics and Computational Biology Group and Center for Excellence in Bioinformatics, René Rachou Institute, Fundação Oswaldo Cruz, Av Augusto de Lima 1715, Belo Horizonte, MG, 30190-002, Brazil

## Abstract

**Background:**

Targeted re-sequencing is one of the most powerful and widely used strategies for population genetics studies because it allows an unbiased screening for variation that is suitable for a wide variety of organisms. Examples of studies that require re-sequencing data are evolutionary inferences, epidemiological studies designed to capture rare polymorphisms responsible for complex traits and screenings for mutations in families and small populations with high incidences of specific genetic diseases. Despite the advent of next-generation sequencing technologies, Sanger sequencing is still the most popular approach in population genetics studies because of the widespread availability of automatic sequencers based on capillary electrophoresis and because it is still less prone to sequencing errors, which is critical in population genetics studies. Two popular software applications for re-sequencing studies are Phred-Phrap-Consed-Polyphred, which performs base calling, alignment, graphical edition and genotype calling and DNAsp, which performs a set of population genetics analyses. These independent tools are the start and end points of basic analyses. In between the use of these tools, there is a set of basic but error-prone tasks to be performed with re-sequencing data.

**Results:**

In order to assist with these intermediate tasks, we developed a pipeline that facilitates data handling typical of re-sequencing studies. Our pipeline: (1) consolidates different outputs produced by distinct Phred-Phrap-Consed contigs sharing a reference sequence; (2) checks for genotyping inconsistencies; (3) reformats genotyping data produced by Polyphred into a matrix of genotypes with individuals as rows and segregating sites as columns; (4) prepares input files for haplotype inferences using the popular software PHASE; and (5) handles PHASE output files that contain only polymorphic sites to reconstruct the inferred haplotypes including polymorphic and monomorphic sites as required by population genetics software for re-sequencing data such as DNAsp.

**Conclusion:**

We tested the pipeline in re-sequencing studies of haploid and diploid data in humans, plants, animals and microorganisms and observed that it allowed a substantial decrease in the time required for sequencing analyses, as well as being a more controlled process that eliminates several classes of error that may occur when handling datasets. The pipeline is also useful for investigators using other tools for sequencing and population genetics analyses.

## Background

Targeted re-sequencing is one of the most powerful and widely used strategies for population genetics studies because it allows screening of variation in a way that is unbiased in respect to the allele frequency spectrum and because it is suitable for a wide variety of living organisms. Although there is a plethora of new opportunities from next-generation sequencing (NGS) technologies [1], re-sequencing studies are traditionally performed using Sanger DNA sequencing. This is due, in part, to the widespread availability of automatic sequencers based on capillary electrophoresis and also to the fact that Sanger sequencing is still less prone to base-calling errors [2], which is critical in population genetics studies for which the accurate identification of substitutions carried by unique chromosomes (singletons) is highly informative [3]. Examples of studies in different areas of genetics that require re-sequencing data are: (a) inferences of past demographic parameters of populations of humans [4,5], animals [6], plants [7] and microorganisms [8], and of the action of natural selection based on ascertainment-bias-free allelic spectra [9-12]; (b) epidemiological studies designed to capture rare polymorphisms responsible for complex traits [13-15]; (c) screening for variation in populations that are not included in public databases such as HapMap, to optimally select informative single nucleotide polymorphism SNPs (tag-SNPs) for association studies [16]; (d) forensic studies or analyses based on mitochondrial DNA data [17,18]; and (e) screenings for mutations in families or small populations with high incidences of specific genetic diseases [19]. Two of the most popular, powerful and freely available tools for re-sequencing studies are (1) the software package Phred-Phrap-Consed-Polyphred (PPCP) [20-24] that performs base calling, alignment, graphical edition and polymorphism identification and (2) the DNA Sequence Polymorphism software (DNAsp) [25], which performs a wide set of population genetics analyses through a user-friendly Windows interface. As these tools were created by different groups, they are not integrated, despite their wide combined use. Frequently, they are the start and end points of basic analyses for many population genetics re-sequencing studies. In between the use of these tools, there are a set of basic but error-prone tasks to be performed with re-sequencing data. In order to facilitate these tasks, we developed and tested a pipeline that improves the handling of sequencing data. Our pipeline was created with the wide community of investigators using PPCP and DNAsp in mind but it is also useful for investigators who use other population genetics packages, such as VariScan [26], the command-line-based version of DNAsp that is designed for large-scale datasets. Forthcoming versions of our pipeline will be integrated with forthcoming Phred-Phrap functions to analyse NGS data and with other computationally robust population genetics tools, such as the libsequence library (http://molpopgen.org/software/libsequence.html[27]).

**Figure 1:**
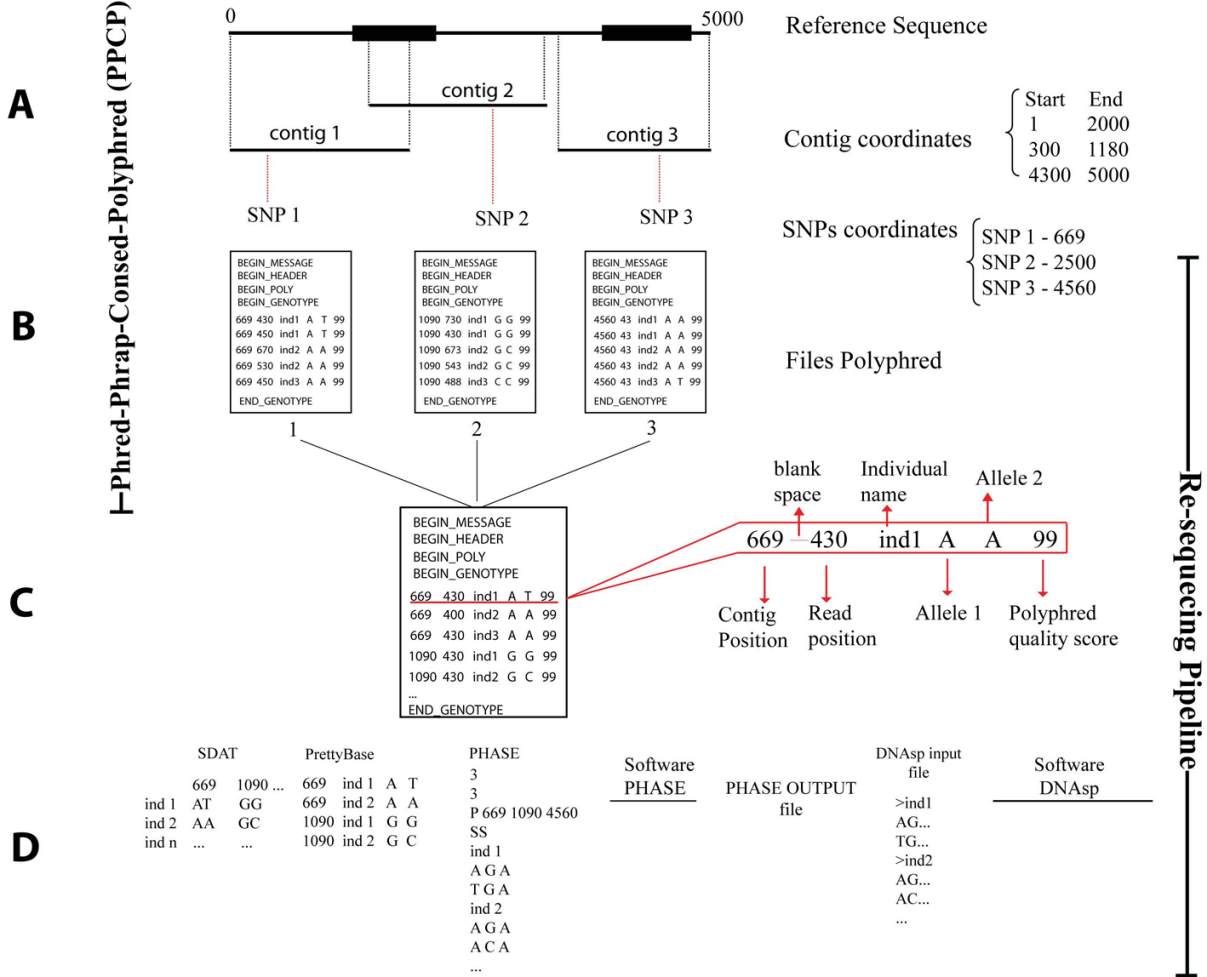
**Overview of the re-sequencing data analysis process integrated with the pipeline**. An example of the entire process of re-sequencing analysis for a specific genomic region from a set of individuals. (A) Re-sequencing steps, base calling, alignment and heterozygous site identification for an entire region sharing a reference sequence. (B) PolyPhred output files of discontinuous sub-regions, such as exons, re-sequenced independently. (C) Consolidation of different exons of a gene that were independently amplified and re-sequenced using the same reference genomic sequence. (D) Files formats that can be handled by the pipeline either as input or output files.

We assume the case of an investigator who is partially or totally re-sequencing a specific genomic region in a set of individuals and that a reference sequence is available for this targeted region (Figure 1). After experimentally obtaining the re-sequencing data (usually with a minimal individual coverage of 2× using forward and reverse primers), the sequencing analyses are performed with software such as PPC. For our purposes (population genetics studies), we define a contig as set of aligned sequences obtained from a set of individuals using the same sequencing primer or a pair of forward/reverse sequencing primers (Figure 1) with a minimum individual coverage of 2× for each sequenced base. In conjunction with PPC, Polyphred is frequently used to automatically identify polymorphic sites and to call genotypes for each read but, in our experience [9,28-30], visual inspection of peaks is necessary to ensure high quality data. After data production and application of quality control (QC) filters (for example, based on Phred scores), the following information should be available for entry into the pipeline: (1) the sequenced regions defined by their coordinates with respect to the reference sequence; and (2) for these regions, the coordinates of the observed segregating sites and their observed genotypes for each read. The pipeline assumes that this information is available in the output format of Polyphred (the Polyphred output file generated for each contig).

## Method

### Design and building

The pipeline was developed as an online system using the Perl programming language for handling dynamic scripting. The current version runs on a Linux/Apache Web server. In order to guarantee portability and accessibility, the system was fully tested in different operating systems and web browsers (see Availability and requirements section).

**Figure 2:**
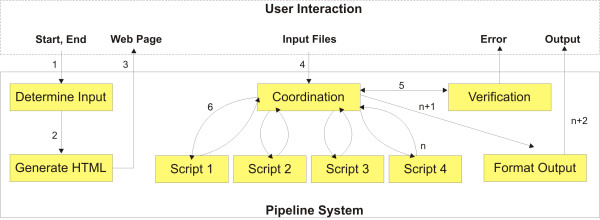
**The system's background operation and user interaction**. The arrows represent the flow of data and controls across the system's modules (boxes) and are labelled according to their order of execution. The system starts by receiving the user's choice of start and end points for the pipeline which represent, respectively, the type of input file that the user has and the format into which the user wants to transform the original file. In accordance with the combination of these start and end points, the system determines the input files (module 'Determine Input') that the user needs to provide in order to complete the chosen path through the pipeline. The required input files are presented to the user as a Web page tailored by the 'Generate HTML' module. The user can then upload the input files that need to be converted to the format required for a specific population genetics program. These files are received by the system's 'Coordination Module', which controls the execution of all required steps through the pipeline, including a verification step (the 'Verification module') for checking whether the provided input files are in their correct formats. Depending on the combination chosen by the user for start and end points, different scripts are invoked by the 'Coordination Module'. These scripts generate outputs that are presented to the user through the 'Format Output Module'.

An overview of the web-based system's architecture is shown in Figure 2. The arrows represent the flow of data and controls across the system's modules (boxes in Figure 2) and are labelled according to their order of execution. The system starts by receiving the user's choice of start and end points for the pipeline which represent, respectively, the type of input file that the user has and the format into which the user wants to transform the original file. In accordance with the combination of these start and end points, the system determines the input files (module 'Determine Input') that the user needs to provide in order to complete the chosen path through the pipeline. The required input files are presented to the user as a Web page tailored by the 'Generate HTML' module. The user can then upload the input files that he or she wants to convert to the format required for a specific population genetics program. These files are received by the system's 'Coordination Module', which controls the execution of all required steps through the pipeline, including a verification step (the 'Verification module') for checking whether the provided input files are in their correct formats. Depending on the combination chosen by the user for start and end points, different scripts are invoked by the 'Coordination Module' (as illustrated in Figure 2). Each script has a specific functionality that is related to a determined file transformation procedure. It is important to note that the modular design of the system's architecture is intended to facilitate future extensions of the pipeline to include other functionalities.

### Web interface

**Figure 3:**
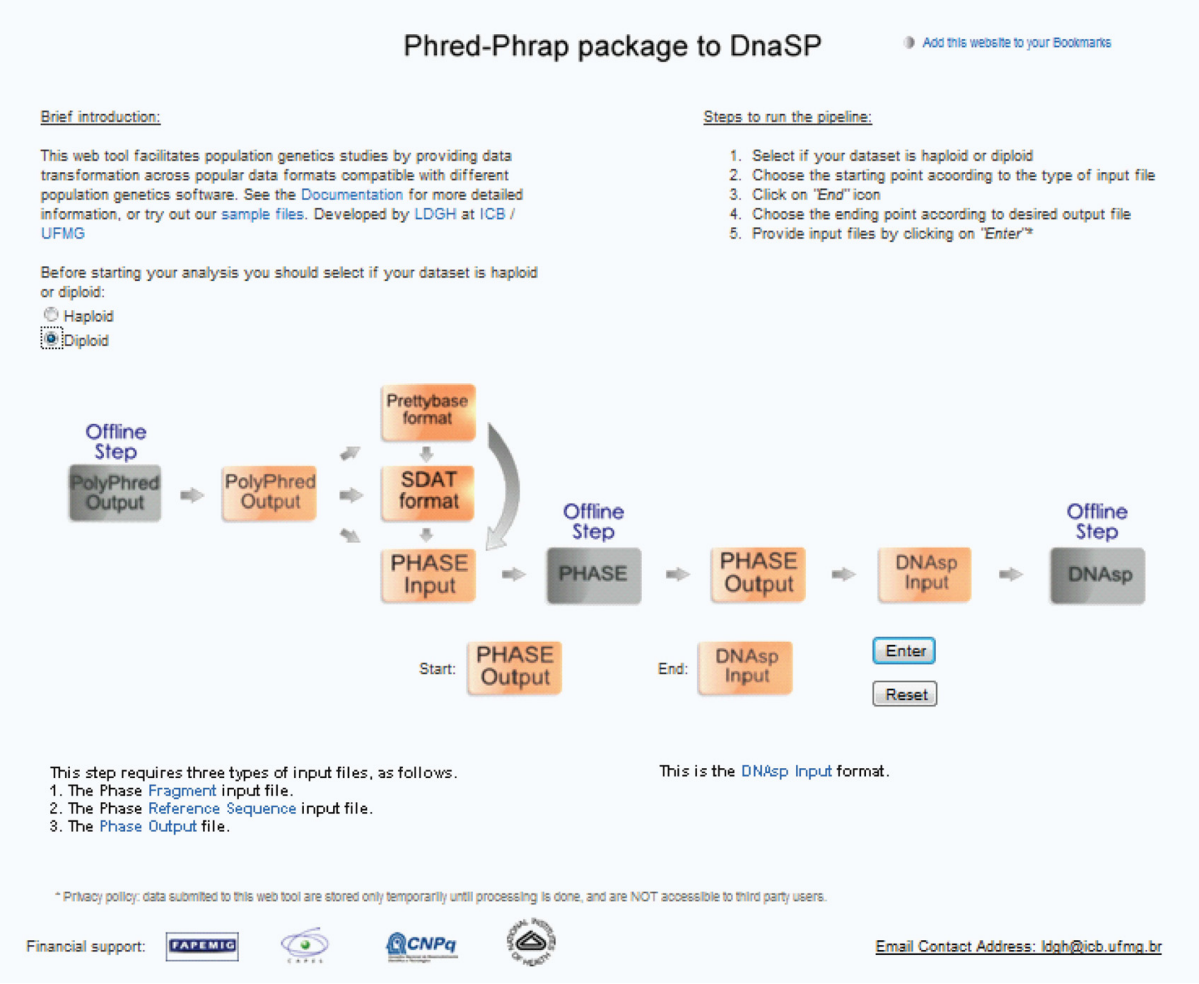
**A snapshot of the main pipeline's Web interface**. The user-friendly interface allows the user to select the desired input and output file formats by clicking within the rectangles (modules) composing the pipeline. The Web page includes a brief introduction to the pipeline with access to end-user documentation and a description of the basic steps needed to run the pipeline. A contact email address is provided in order to guarantee permanent user support.

The system's external shell, behind which lies the described architecture, is the web interface illustrated in Figure 3. The grey rectangles in Figure 3 represent the steps of the pipeline that are not automated, such as PHASE and DNAsp executions. The light coloured rectangles represent the modules or functionalities provided by the pipeline, which can be combined in order to reach the desired output. The user-friendly interface allows the user to select the desired start and end points of the pipeline by clicking within the rectangles (or modules) composing the pipeline. Whenever the user clicks on one of the rectangles, a brief explanation of the type of input file that it accepts and the output file that it generates is shown. The system then indicates the input files that need to be provided by the user in order to run the chosen path through the pipeline. This is performed dynamically depending on the user's choice of start and end points. After the selection of the start and end points, no user intervention is needed until the final output is presented.

## Results

The web interface of the pipeline is shown in Figure 3. The pipeline allows the procedures described below to be performed using a web page with a graphical and user-friendly interface http://www.cebio.org/pipelineldgh. Step 1 integrates different outputs produced by different PPCP contigs that share a reference sequence (Figure 1B). For instance, this step can combine different exons of a gene that have been independently amplified and re-sequenced, so that they might be analysed using a shared reference genomic sequence (Figure 1C). Step 2 reformats genotypes from reads in Polyphred output file format into a user-friendly rectangular matrix of genotypes with individuals as rows and segregating sites as columns (for example, SDAT format; Figure 1D). In this step, the pipeline consolidates reads from the same individuals (sharing the same identifier) by checking for genotype inconsistencies among different reads of the same individual (for example, forward and reverse reads of the same amplicon). In the case of diploid data, if the investigator prefers to infer haplotypes using the popular software PHASE [31], which requires multiple runs with specific parameters, the pipeline prepares the input files for PHASE (Step 3; Figure 1D). PHASE output files contain the inferred haplotypes for each individual but only include the segregating sites. For some population genetics analyses using re-sequencing data (for example, DNAsp), it is necessary to reconstruct the entire sequence, including both monomorphic and polymorphic sites. Step 4 of the pipeline uses the reference sequence and the information from PHASE output files (positions of segregating sites in relation to the reference sequences and inferred haplotypes for each individual) to reconstruct for the targeted region the two DNA sequences corresponding to the two inferred haplotypes of each individual. The pipeline generates a FASTA file that may be used as input for DNAsp or other population genetics tools (Figure 1D).

## Discussion

The following are features of the pipeline that deserve additional commentary.

### Data production and the use of the pipeline

There are different experimental approaches to the generation data for a re-sequencing population genetics project. It is possible to continuously re-sequence an entire region or to target specific discontinuous subregions, such as exons (Figure 1A). In order to achieve these goals, different strategies that combine polymerase chain reaction (PCR) and re-sequencing are available. For instance, it is possible to amplify regions of ~400-600 bps that will be independently re-sequenced [32]. It is also possible to amplify larger regions consisting of a few kilobases by long-PCR [28] and to perform more than one re-sequencing reaction on each amplicon. In our experience, independent of the wet-lab strategy, two procedures are advisable to analyse the sequencing data. First, we recommend the use of a unique reference sequence for the entire genomic region, which allows unambiguous determination of the position of variable sites independently of their position on each read. Second, each set of reads that is re-sequenced using the same sequencing primers (or with forward and reverse primers) should be aligned separately (such as, in different Phrap-Consed contigs). These procedures minimize the mix of good and bad quality calls for a specific position in the same contig, which facilitates both automatic and visual genotype calls.

When using PPCP to analyse reads in small- to medium-scale re-sequencing studies, we perform visual verification of the chromatograms. Although Polyphred genotype calls are very useful, the process is prone to mistakes, particularly for heterozygous genotypes. We observed that this miscalling happens in around 2.5% of genotype calls (in 15% of the inferred SNPs), considering good quality reads (phred scores > 30) and data generated with *Applied Biosystems *BigDye v.3.1 and run in a 3730 or 3100 *Applied Biosystems *sequencer (calculated from unpublished data from ETS and SJC on the basis of ~7 Mb re-sequenced in a population genetics study). For this reason, we visually check all *Consed *chromatogram peaks that are both monomorphic (called by Phred) and polymorphic (called by Polyphred).

### Haploid data

Our pipeline was developed keeping in mind the more general case of diploid data. However, it may be easily used with haploid data. There is an option to specify if the data to be analysed are haploid or diploid and conveniently adapting outputs to this information. We recommend that users interested in analysing haploid data follow the same procedures specified for the analysis of diploid data, assuming that all genotypes are homozygous.

### Haplotype inferences using PHASE

Although the latest version of DNAsp (v. 5.0) incorporates the algorithm implemented in the PHASE software [31], investigators may prefer running PHASE separately for several reasons: the need to use different parameters for burn-in and length of the runs; the possibility of performing the computationally demanding haplotype inferences in a more powerful computer; or the preference for the PHASE for Linux/Unix platforms, which bypasses the limitations of the Windows version. We developed the pipeline with the user who prefers to run PHASE separately in mind. However, for large datasets, inferences using PHASE may be computationally prohibitive. In this case, a faster, although less accurate method, was implemented using the software fastPHASE [33]. As input files for fastPHASE and PHASE are the same, our pipeline is compatible with both programs.

### QC procedures of the pipeline

In order to save time preparing input files, our pipeline has a set of QC procedures that are executed before any of the file formatting steps is performed. This includes the identification of inconsistent genotype calls for different reads of the same individuals and the verification of the different input/output files' formats.

### Future developments

We will continue to expand the functions of our pipeline, so that it will include: (a) reformatting of SDAT files to generate a Haploview input file for linkage disequilibrium analysis; (b) the option of reformatting files in both directions (for example, being able to generate the Polyphred output from the SDAT format; and (c) the possibility of generating either the SDAT file format or the DNAsp FASTA file for diploid organisms using the International Union of Pure and Applied Chemistry ambiguity nomenclature for heterozygous genotypes.

## Conclusions

Our pipeline is designed to handle re-sequencing data and is complementary to resources such as FORMATOMATIC [34] and CONVERT http://www.agriculture.purdue.edu/fnr/html/faculty/rhodes/students%20and%20staff/glaubitz/software.htm, which are useful for analysing microsatellites and SNPs but not for sequencing data. We tested our pipeline with several users who were performing re-sequencing studies of haploid and diploid loci in humans, plants, animals and microorganisms. We verified that our pipeline is robust and substantially decreases the time required for re-sequencing data analyses. Also, our pipeline allows for a more controlled process that eliminates several classes of error that may occur in population genetics, epidemiological, clinical and forensic studies when handling such data.

## Availability and requirements

The sequencing pipeline is available at http://www.cebio.org/pipelineldgh.

The web-based system will be freely available for academic purposes.

Operating systems: Windows, 32-bit Linux, 64-bit Linux, MAC-OS.

Programming languages: Perl, HTML and JavaScript.

Browsers: Internet Explorer (Windows), Firefox (Linux, Windows), Safari (MAC-OS)

## Abbreviations

DNAsp: DNA sequence polymorphism; NGS: next generation sequencing; PCR: polymerase chain reaction; PPCP: Phred-Phrap-Consed-Polyphred; QC: quality control; SNP: single nucleotide polymorphism.

## Competing interests

The authors declare that they have no competing interests.

## Authors' contributions

The first two authors MM and WCSM contributed equally to the paper. ETS conceived the project. WCSM, AS, ETS and BA developed the scripts used in this work. MM tested different versions and parts of the pipeline, interacted with several investigators and research groups and wrote the Web service documentation. AS, BA, WCSM and MR designed and integrated the pipeline modules and developed the Web interface. ETS and MR supervised the project. SJC provided resources and participated during the early parts of the project. LS provided the resources for hosting and maintaining the Web interface under the supervision of GO. ETS, MR and WCSM wrote the manuscript. All the authors read and approved the final manuscript.
